# Transmission of Brucellosis from Elk to Cattle and Bison, Greater Yellowstone Area, USA, 2002–2012

**DOI:** 10.3201/eid1912.130167

**Published:** 2013-12

**Authors:** Jack C. Rhyan, Pauline Nol, Christine Quance, Arnold Gertonson, John Belfrage, Lauren Harris, Kelly Straka, Suelee Robbe-Austerman

**Affiliations:** National Wildlife Research Center, Fort Collins, Colorado, USA (J.C. Rhyan, P. Nol, L. Harris, K. Straka);; Natural Resources Research Center, Fort Collins (A. Gertonson, J. Belfrage);; National Veterinary Services Laboratories, Ames, Iowa, USA (C. Quance, S. Robbe-Austerman)

**Keywords:** brucellosis, wildlife, livestock, epidemiology, elk, bison, cattle, Idaho, Wyoming, Montana, Greater Yellowstone Area, bacteria, zoonoses

## Abstract

Bovine brucellosis has been nearly eliminated from livestock in the United States. Bison and elk in the Greater Yellowstone Area remain reservoirs for the disease. During 1990–2002, no known cases occurred in Greater Yellowstone Area livestock. Since then, 17 transmission events from wildlife to livestock have been investigated.

Bovine brucellosis, caused by *Brucella abortus*, is a global zoonotic disease primarily infecting cattle, in which it produces abortions, retained placentas, male reproductive tract lesions, arthritis, and bursitis. In humans, brucellosis can cause recurrent fever, night sweats, joint and back pain, other influenza-like symptoms, and arthritis. In animals and humans, it can persist for long periods. During the 1930s, a state–federal cooperative effort was begun to eliminate the disease from livestock in the United States. From an initial estimated prevalence in 1934 of ≈15%, with nearly 50% of cattle herds having evidence of infection (*1*,*2*), the United States now has no known infected livestock herds outside of portions of Idaho, Wyoming, and Montana, adjacent to Grand Teton and Yellowstone National Parks. This area, referred to as the Greater Yellowstone Area (GYA), also encompasses state and federal feeding grounds in Wyoming where elk are fed during the winter. Considered a spillover disease from cattle to elk and bison, brucellosis now regularly spills back from elk to cattle. Although bison-to-cattle transmission has been demonstrated experimentally and in nature (*3*,*4*), it has not been reported in the GYA, probably because of ongoing rigorous management actions to keep cattle and bison spatially and temporally separated.

In 1992, a court case highlighted the potential for transmission of brucellosis from free-ranging wildlife to livestock in the GYA. The litigation concerned brucellosis transmission purportedly from elk or bison to 2 cattle herds in 1988 and 1989 (*5*). Before those incidents and since ≈1961, brucellosis had been detected in 4 GYA cattle herds, and transmission was attributed to a wildlife source on the basis of epidemiologic investigations (*6*). From 1990 through 2001, no brucellosis was found in any GYA livestock despite intensive surveillance in some areas, precipitated by court action. We report a series of recent cases in which brucellosis was transmitted from free-ranging elk to domestic cattle or ranched bison as determined by epidemiologic and microbiological investigations.

## The Study

**Figure 1:**
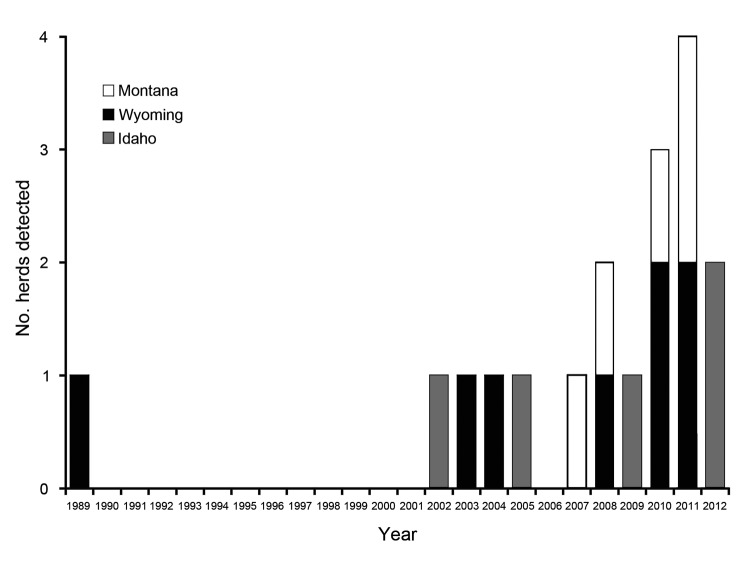
Number of *Brucella abortus*–positive domestic cattle and ranched bison herds (combined) detected each year, Greater Yellowstone Area, USA, 1989–2012.

During April 2002–April 2012, brucellosis was discovered in 13 beef cattle herds and 4 ranched bison herds in the GYA (Figure 1). Additionally, from comingling of cattle herds at the time of transmission and transfer of ownership of some animals between infection and detection, 3 more infected cattle herds were identified. In each of the 17 herds, infection was detected by serologic testing and confirmed by culture of tissues collected at slaughter of >1 animals. The source of infection of each cattle or bison herd was determined through extensive epidemiologic investigations by state and federal animal health authorities. These investigations included serologic testing of all cattle herds adjacent to or in contact with the infected herd, testing of all herds from which the infected herds had received animals in the preceding years, interviews with owners and managers to determine the history of comingling with wildlife, and comparison of DNA test results of the isolates with those from wildlife and domestic animals. The transmission event in 2002 was previously reported (*7*).

We subjected 248 *B. abortus* isolates from affected cattle and bison herds and surrounding wild elk and bison to a 10-loci variable number tandem repeat assay. We analyzed results using a minimum spanning tree model (*8*).

Cattle and bison herd sizes varied from <50 to >300 animals, and seroprevalence ranged from 0.2% to 20% (Table). For 8 herds, infection initially was detected through required testing of cattle going through markets or to slaughter. Four infected herds were detected by routine testing because of the location of herds in the brucellosis-endemic area, 1 herd was detected because of testing of area herds in proximity to a previously infected herd, and 4 herds were detected by testing required for interstate transport or change of ownership.

**Table:** Cattle and ranched bison herds found infected with *Brucella abortus* due to transmission from elk, Greater Yellowstone Area, USA

Herd no.	County, state	Species	Herd size	Date detected	Seropositive, %	Culture results	Distance to feeding ground, km
1	Fremont, ID	Cattle	50–100	2002 Apr	12.0	Biovar 1	50*
2	Sublette, WY	Cattle	>300	2003 Oct	9.9	Biovar 1	2.4
3†	Teton, WY	Cattle	>300	2004 Jun	1.9	Biovar 4	Adjacent
4	Bonneville, ID	Cattle	<50	2005 Aug	20.0	Biovar 1	85‡
5	Park§, MT	Cattle	>300	2007 May	0.2	Biovar 1	>100
6	Park, MT	Cattle	<50	2008 May	2.9	Biovar 1	>100
7	Sublette, WY	Cattle	>300	2008 Jun	5.5	Biovar 4	24
8	Jefferson, ID	Cattle	>300	2009 Jul	1.5	Biovar 1	85
9	Park, WY	Cattle	>300	2010 Oct	1.1	Biovar 1	>100
10	Park, WY	Bison	200–300	2010 Nov	11.5	Biovar 4	>100
12	Park, WY	Cattle	>300	2011 Feb	0.9	Biovar 1	>100
13	Park, WY	Cattle	>300	2011 Sep	1.2	Biovar 1	>100
14	Park, MT	Cattle	>300	2011 Sep	2.0	Biovar 1	>100
15	Madison, MT	Bison	>300	2011 Nov	0.2	Biovar 1	>100
16	Fremont, ID	Cattle	50–100	2012 Apr	5.8	Biovar 1	90
17	Bonneville, ID	Bison	200–300	2012 Mar	0.7	Biovar 4	40

*Elk were also intentionally fed on ranch by owner. †Brucellosis was detected in 2 herds that were pastured together during spring of 2004. This was considered a single transmission event, and statistics are given for the combined herds. ‡Herd located 0.8 km from site where elk feeding ground had been until 2003. §This herd was discovered infected in Carbon County, MT, in 2007, but animals had been transported from Park County in 2005. Index cow aborted in 2005 and did not calve in 2006.

## Conclusions

**Figure 2:**
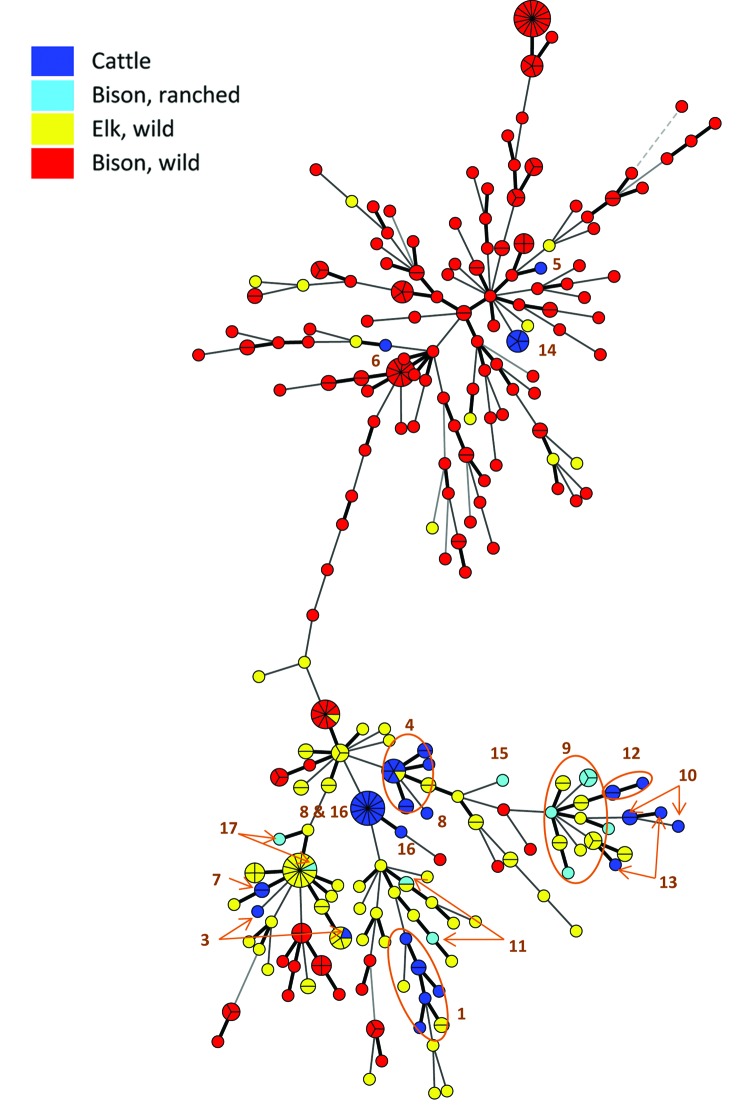
Minimum spanning tree generated from variable number tandem repeat (VNTR) data for 348 *Brucella abortus* isolates in the National Veterinary Services Laboratory database. Each sphere, or node, represents a unique VNTR type. Nodes are color coded according to the source of the isolate, and segments of nodes represent isolates from different animals with the same VNTR profile. The numbers represent the herd designations as indicated in the Table (note that herd no. 2 is not represented in this figure).

Examination of the data from these herds reveals several facts. With few exceptions, herds had low seroprevalence at time of detection (14 of 17 herds: <10%; 11 of 17 herds: <3%). Additionally, few or no abortions were reported by herd owners or managers. These findings probably are due to rigorous surveillance and the widespread use of vaccination in GYA herds. The attenuated live vaccine, strain RB51, is efficacious in decreasing abortions but does not prevent infection (*9*). The herd with highest seroprevalence (herd no. 4) had the lowest percentage of vaccinated cattle (41%). Nearly all animals in other herds had received calfhood vaccination. In several cases, management actions may have increased risk for exposure (i.e., allowing elk to feed with cattle and placing cattle in pasture with elk during late winter or spring). In Wyoming and Idaho, proximity to elk feeding grounds varied. With 1 exception (herd no. 7), owners or investigators reported that elk were sharing the premises with cattle. Herd no. 7’s owner and his son were employed at a nearby elk feeding ground, where their duties included removal of elk fetuses, which created the potential for transmission through fomites. Information obtained from state wildlife agencies indicated that in every transmission case, serologic surveillance of elk in the area showed some level of infection in that species. On the basis of the 10-loci variable number tandem repeat assay, the *B. abortus* isolates recovered from cattle and farmed bison are very closely related to—and sometimes indistinguishable from—isolates from wild elk (Figure 2).

Seventeen instances of brucellosis transmission from elk to livestock were reported during the last decade. This crescendo of interspecies transmission in all 3 GYA states and involving ranches in proximity to and remote from elk feeding grounds suggests a change or combination of changes in risk factors in the GYA ecosystem. Until the discovery of increasing prevalence in non–feeding ground elk (2006–2008) (*10*), *B. abortus* infection was not believed to have been self-sustaining in these populations (*6*). This belief was supported by high seroprevalence in populations in proximity to feeding grounds, with a marked decrease in prevalence proportional to distance from feeding grounds. In the last decade, however, seroprevalence in some non–feeding ground elk herds has increased to levels similar to those of feeding ground herds, suggesting that brucellosis is now self-sustaining in these populations (*11*).

Several factors are likely to have contributed to changes in elk distributions and the resulting increases in brucellosis in some populations and its transmission to livestock. These include population and density increases (*11*,*12*), changes in land management that created safe havens for elk (*13*), and reintroduction of wolves to the GYA (*14*). Other factors that might have had local or general effects include climatic and snowfall changes (*15*), reduction of habitat by urbanization, and increased use of motorized backcountry vehicles. Changes in elk and cattle brucellosis surveillance during the last decade do not account for the disease spread in elk or increased transmission to livestock.

If brucellosis continues to increase among free-ranging elk populations remote from feeding grounds, the area to which brucellosis is endemic is likely to expand and the risk for transmission to livestock and the public will increase, in part reversing the hard-fought gains of the past 75 years in eliminating the disease in the United States. Gaining a better understanding of ecologic and sociologic changes in the GYA and their impact on the epidemiology of this wildlife–livestock–human interface disease is essential to developing effective management strategies.
